# Learning misclassification costs for imbalanced classification on gene expression data

**DOI:** 10.1186/s12859-019-3255-x

**Published:** 2019-12-24

**Authors:** Huijuan Lu, Yige Xu, Minchao Ye, Ke Yan, Zhigang Gao, Qun Jin

**Affiliations:** 1 Key Laboratory of Electromagnetic Wave Information Technology and Metrology of Zhejiang Province, College of Information Engineering, China Jiliang University, Hangzhou, China; 2 College of Computer Science and Technology, Hangzhou Dianzi University, Hangzhou, China; 3 Faculty of Human Sciences, Waseda University, Tokorozawa, Japan

**Keywords:** Cost-sensitive, Misclassification cost, Weighted classification accuracy, Parameter fitting

## Abstract

**Background:**

Cost-sensitive algorithm is an effective strategy to solve imbalanced classification problem. However, the misclassification costs are usually determined empirically based on user expertise, which leads to unstable performance of cost-sensitive classification. Therefore, an efficient and accurate method is needed to calculate the optimal cost weights.

**Results:**

In this paper, two approaches are proposed to search for the optimal cost weights, targeting at the highest weighted classification accuracy (WCA). One is the optimal cost weights grid searching and the other is the function fitting. Comparisons are made between these between the two algorithms above. In experiments, we classify imbalanced gene expression data using extreme learning machine to test the cost weights obtained by the two approaches.

**Conclusions:**

Comprehensive experimental results show that the function fitting method is generally more efficient, which can well find the optimal cost weights with acceptable WCA.

## Background

Classification of gene expression data reveals tremendous information in various application fields of biomedical research, such as cancer diagnosis, prognosis and predictions [1–3]. However, the gene expression data is composed of high-dimensional, noisy and imbalanced data samples [4]. The characteristic of imbalanced data is serious imbalance in the proportion of positive and negative samples [5, 6]. Gene expression data exacts a series of pre-processing steps to eliminate misleading classification results [7]. Moreover, the classification of gene expression data is a cost-sensitive problem, although both positive and negative classifications of cancer genes provide important evidences for doctors to make the treatment plan.

Traditional machine learning algorithms usually assume that the training set is balanced. For imbalanced datasets, such as the gene expression datasets, the classical classification algorithms with the correct classification rates (CCR) may bias towards the majority classes. However, the misclassifications of minority classes usually contribute the higher influences than those of majority classes. Therefore, The introduction of cost sensitive learning (CSL) is necessary to eliminate the defects of traditional classification algorithms for imbalanced datasets. Traditionally, oversampling the minority class, undersampling the majority class, and synthesizing new minority classes can be used to handle this problem. In this work, we utilize a more sophisticated way to search for the optimal weights, and the proposed methods are more advanced than ever.

In CSL, misclassification cost is an important factor to evaluate the classification performance of imbalanced datasets. However, solving the misclassification cost matrix is not a trivial task in many situations [8–10]. A direct solution for finding the misclassification costs is to assign them manually according to user expertise or inversely calculate the costs based on class distribution [11–13]. More sophisticated solutions can be found by fitting the importance of features to adaptive equations.

In this paper, we learn the misclassification cost from the evaluation functions of cost-sensitive algorithms, using weighted classification accuracy as the measurement of cost-sensitive classification performance. The cost weights that lead to optimal classification performance are learned by grid searching strategy. It will help the researchers to obtain a reference weight. Then, three fitting functions will be found to represent the optimal cost weights. A series of comprehensive experimental results show that the function fitting approach is an effective way of finding the optimal cost weights, targeting at high weighted classification accuracy (WCA). Fitting functions can accurately locate optimal weights. Appropriate weights will greatly improve the accuracy of the model.

Imbalanced data greatly affects the accuracy of classification. We discuss the cost-sensitive classification algorithms in the imbalance problem. CSL is one of the most hot topics in the field of machine learning. Many works have studied on CSL and embedded the misclassification costs into various classifiers, such as the decision trees (DTs), support vector machines (SVMs) and extreme learning machines (ELMs). Chai et al. [14] considered the testing costs of missing values in naive Bayes (NB) and DT algorithms. Feng [15] defined a customized objective function for misclassification costs and designed a score evaluation based cost-sensitive DT. For multi-class classification problems, Feng’s method generally achieves higher classification accuracy or lower misclassification costs. Zhao and Li [16] extended the evaluation function by including weighted information gain ratio and the test cost for the cost-sensitive DT. The proposed cost-sensitive DT algorithm not only reduced the misclassification cost, but also improved the classification efficiency of the original C4.5 algorithm [17, 18]. Lu et al. [19] made use of the cost-sensitive DTs as base classifiers and constructed a cost-sensitive rotational forest. Two kinds of DTs, i.e., EG2 and C4.5, are considered and tested [20]. These experiments show that integrating cost-sensitive to classification algorithms can effectively improve classification efficiency.

Cost sensitivity and classification algorithms combine to form efficient classification methods. Cao et al. [21] proposed to embed evaluation measures into the objective function for to improve the performance of a cost-sensitive support vector machine (CS-SVM). He et al. [22] integrated the Gaussian Mixture Model (GMM) into the CS-SVM to deal with the imbalanced classification problem. Cheng and Wu [23] added weights to features and introduced a weighted features cost-sensitive SVM (WF-CSSVM). The WF-CSSVM algorithm showed significant performance improvement on both aspects of accuracy and cost. Silva et al. [24] combined CS-SVM with semi-supervised learning method to form a hybrid classification algorithm. The effectiveness of the proposed hybrid method is shown in the experimental results on Earth monitoring and landscape mapping. Cao et al. [25] tackled the problem of multi-labeled imbalanced data classification problem. They successfully assigned different misclassification costs to different label sets for reducing the overall misclassification cost.

CS-ELM has been studied by many researchers in various aspects. Zong et al. [26] introduced a weighted extreme learning machine (WELM) for imbalanced data learning. It was claimed that the WELM can be extended to a cost-sensitive ELM (CS-ELM). Zheng et al. [27] formally applied the concept of the cost-sensitivity to extreme learning machine (ELM). Yan et al. [28, 29] extended Zheng et al.’s work and introduced a cost-sensitive dissimilar ELM (CS-D-ELM). Compared to traditional ELM algorithms, the CS-ELM algorithms guarantee the classification accuracy and reduce the misclassification cost. More recently, Zhang and Zhang [30] solved the problem of defining and optimizing the cost matrix for CS-ELM to make it more robust and stable [31, 32]. Zhu and Wang [33] treated CS-ELM as a base classifier to solve a semi-supervised learning problem. Incremental results show that the CS-ELM has better performance in terms of accuracy, cost, efficiency and robustness over other existing classifiers.

### Classical definition of cost matrix

Considering the binary classification problem, the confusion matrix shows four types of classification results according to the prediction values, namely, true positive, false positive, false negative and true negative (Table 1) [34, 35].

**Table 1:** The confusion matrix for binary classification

	Prediction of Positive	Prediction of Negative
Positive samples	True Positive TP	False Negative FN
Negative samples	False Positive FP	True Negative TN

The CSL seeks the overall minimum cost by introducing sensitive costs, rather than only aiming at high CCR. While there are several types of classification costs, it should be noted that this work only focuses on the misclassification cost.

Misclassification cost can be viewed as penalties for errors in the classification process. In binary classification problems, costs caused by different types of errors may be different. We define the minority class as positive (*P*), the majority class as negative (*N*), and construct the cost matrix *C* as shown in Table 2.

**Table 2:** Cost matrix

Predicted Actual	*P*	*N*
*P*	*C*_*00*_	*C*_*01*_
*N*	*C*_*10*_	*C*_*11*_

In Table 2, *C*_*00*_ and *C*_*11*_ show the cost of correct classification. By default, we set the costs of correct classifications as 0. *C*_*01*_ and *C*_*10*_ show the costs of error classifications, where *C*_*01*_ denotes the misclassification costs of samples from *P* class, and *C*_*10*_ denotes the misclassification costs of samples from *N* class. Therefore, the cost matrix in Table 2 can be simplified as:

1 $$ C=\left[\begin{array}{cc}0& {C}_{01}\\ {}{C}_{10}& 0\end{array}\right] $$

### Correct classification rates versus weighted classification accuracy

For classical machine learning problems, the classification accuracy always refers to the correct classification rate (CCR) [36–38], or called overall accuracy (OA) [39–42], which is the proportion of all correctly classified samples:

2 $$ OA=\frac{TP+ TN}{TP+ FN+ TN+ FP}\times 100\% $$

However, for imbalanced datasets where the numbers of positive and negative samples differ significantly, the CCR might be misleading [43, 44]. Considering a test set containing 99 negative samples but with only one positive sample [45, 46], a poorly designed classifier that simply puts all samples as negative will achieve an overall accuracy of 99/100 = 0.99, even though the accuracy for positive class is 0. To resolve this issue, we introduce the notion of adaptive classification accuracy (ACA) defined as follows:

3 $$ ACA=\frac{1}{2}\cdot \left(\frac{TP}{TP+ FN}+\frac{TN}{TN+ FP}\right) $$

By embedding a weight *w*_i_ into the *i-*th class, we get the weighted classification accuracy (WCA) as:

4 $$ WCA=\frac{w_1}{w_1+{w}_2}\cdot \frac{TP}{TP+ FN}+\frac{w_2}{w_1+{w}_2}\cdot \frac{TN}{TN+ FP} $$

By enforcing *w*_1_ + *w*_2_ = 1, Formula (9) is reduced to:

5 $$ WCA={w}_1\cdot \frac{TP}{TP+ FN}+{w}_2\cdot \frac{TN}{TN+ FP} $$

Formula (10) can be easily extended to multi-classification problems:

6 $$ WC{A}_n=\sum \limits_{i=1}^n{w}_i\frac{C{M}_i}{M_i},\sum \limits_{i=1}^n{w}_i=1 $$

where *n* denotes the number of classes, *M*_*i*_ (*i = 1, 2,..., n*) denotes the number of samples belonging to the *i*-th class, and *CM*_i_ (*i = 1, 2,..., n*) denotes the number of correctly classified samples within *i*-th class. Since the WCA is more accurate describing the classification accuracy, we use the WCA to evaluate the classification performance of cost-sensitive classifiers in the problem of gene expression data classification.

## Methods

### Optimal cost weights searching

From the University of California Irvine (UCI) standard classification dataset, we choose Leukemia, Colon, Prostate, Lung and Ovarian gene as the datasets for cost weights searching and further test, i.e., the Leukemia cancer dataset, the Colon cancer dataset, the Prostate cancer dataset, the Lung cancer dataset, and the Ovarian cancer in the tumor data respectively. All details of aforementioned datasets are shown in Table 3.

**Table 3:** Specifications of datasets

Dataset	Sample number	Feature dimension	Classification number
Leukemia	34	7130	2
Colon	62	2000	2
Prostate	136	12600	2
Lung	181	12533	2
Ovarian	253	15154	2

### Optimal cost weights searching by grid searching strategy

The optimal weights are searched by an adaptive algorithm using grid searching. There are two crucial factors to consider: the sample importance *w* and sample categorical distribution *p*. The sample categorical distribution *p* is the proportion between the number of positive class and negative class in test sets. Test set is constructed by random sampling. As such, it is necessary to study the relationship between the three factors, namely, *w*, *p* and WCA, where WCA is the fitness value for the grid searching strategy. In general, the grid searching strategy can be described as follows (the detailed algorithm steps are listed in Table 4):

1) Set the searching region as *M*, grid searching step size as *T*, and the initial position as *P*_0_;

2) Calculate the fitness of the current position, record the position *P*_max_ that has the best fitness *f*_max_ (*f*_max_ = WCA);

3) Update current location, *P*=*P* + *T*;

4) if the current fitness value is greater than *f*_max_, update *f*_max_ and *P*_max_;

5) return *f*_max_ and *P*_max._

**Table 4:** Grid Searching Strategy

Grid Searching Strategy	
1: procedure GRIDSEARCHING(*M*, *T*, *P*_0_)	
2: *P* = *P*_0_	
3: *f* = WCA(*P*)	
4: if *P* < *M* then	
5: *P* = *P* + *T*	
6: if *f* > *f*_max_ then	
7: *f*_max_ = *f*	
8: *P*_max_ = *P*	
9: end if	
10: end if	
11: return *P*_max_, *f*_max_	
12: end procedure	

Extreme learning machine is an effective single hidden-layer feed-forward neural network (SLFN) learning algorithm. Cost-sensitive extreme learning machine (CS-ELM) is a kind of ELM, which attaches a cost matrix on output layer. In this research, we set the number of hidden neurons at 10. Less neurons will make the result more sensitive to observe the change of weights. And seven different gene expression datasets are used to obtain the classification results with CS-ELM as the classifier. CS-ELM minimizes the conditional risk by embedding misclassification cost in ELM.

7 $$ argmin\ R\left(i|x\right)= argmin\ \sum \limits_jP\left(j|x\right)\bullet C\left(i,j\right) $$

where *R(i|x)* is the conditional risk when the sample *x* is assigned to the class *i*, and *P(j|x)* is the conditional probability that *x* belongs to *j*, *C(i, j)* is the risk of misclassifying *j* to class *i*, where *i*, *j* ∈ {*c*_1_*, c*_2_*,* …*, c*_m_} and *m* is the number of classification categories.

## Results

### Optimal cost weights searching by function fitting

In this subsection, we use *w* and *p* as independent variables, and define a function fitting problem as:

8 $$ {w}_c=f\left(w,p\right) $$

where *w*_*c*_ = *C*_01_/*C*_10_, *w* = *w*_1_/(*w*_1_ + *w*_2_) and *p* represents the proportion of positive and negative classes. We set *C*_*10*_ to 1 to reduce the complexity of calculation, i.e., *f*_c_ = *C*_*01*_.

**Table 5:** Optimal weights for different data set

Data set	Sample categorical	Influence factors		Optimal weights *w*_c_	WCA
	distribution *p*	*w*_1_/(*w*_1_+*w*_2_)	*w*_2_/(*w*_1_+*w*_2_)		
Colon	1	0.2	0.8	1.03	0.6167
Leukemia	1.33	0.9	0.1	0.9	0.9179
Ovarian1	1.68	0.9	0.1	1.65	0.9055
Prostate1	2	0.9	0.1	1.06	0.939
Prostate2	2.5	0.9	0.1	1.04	0.9372
Lung1	3	0.9	0.1	0.93	0.92
Ovarian2	4	0.1	0.9	3.45	0.9094
Lung2	5	0.1	0.9	4.26	0.9078
Ovarian3	6.5	0.9	0.1	0.8	0.9075
Lung3	8	0.9	0.1	0.92	0.9009

The sample distribution *p*, the optimal weight *w*_*c*_ = *C*_01_/*C*_10_ and the highest fitness value of each dataset are listed in Table 6.

**Table 6:** Datasets, cost weights and WCAs with the two approaches proposed

Dataset			Cost weight				WCA				
type	*p*	*w*	optimal	*w*_*c1*_	*w*_*c2*_	*w*_*c3*_	optimal	*w*_*c1*_	*w*_*c2*_	*w*_*c3*_	*ECSELM*
ovarian	1.68	0.1	1.65	1.63	1.53	1.58	0.9055	0.9695	0.1966	0.2084	0.1017
Prostate	2.5	0.9	1.04	1.05	1.05	1	0.9372	0.9815	0.9509	0.9869	0.8985
Lung1	5	0.1	4.26	4.03	4.1	3.94	0.9078	0.9778	0.9786	0.9779	0.875
Lung2	8	0.9	0.92	0.9	0.66	0.61	0.9009	0.9564	0.9762	0.9675	0.9

We use an automatic fitting software named 1STOPT to do the function fitting [47]. In 1STOPT, Levenberg-Marquardt and Universal Global Optimization are used to fit functions. We compared 500 functions with different types, and selected the three functions with the highest correlation coefficient:

9 $$ {w}_{c1}={f}_1\left(w,p\right)=\frac{a_1+{a}_2\cdot w+{a}_3\cdot {w}^2+{a}_4\cdot {w}^3+{a}_5\cdot {a}_{12}\cdot \ln p+{a}_6\cdot {\left({a}_{12}\cdot \ln p\right)}^2}{1+{a}_7\cdot w+{a}_8\cdot {w}^2+{a}_9\cdot {a}_{12}\cdot \ln p+{a}_{10}\cdot {\left({a}_{12}\cdot \ln p\right)}^2+{a}_{11}\cdot {\left({a}_{12}\cdot \ln p\right)}^3} $$

where *a*_1_ = 1.323, *a*_2_ = − 2.278, *a*_3_ = 3.047, *a*_4_ = − 1.286, *a*_5_ = − 1.746, *a*_6_ = 0.998, *a*_7_ = − 0.400, *a*_8_ = 0.369, *a*_9_ = − 2.606, *a*_10_ = 2.544, *a*_11_ = − 0.818, *a*_12_ = 0.482. The correlation coefficient *R*_*1*_ of *f*_*1*_ is 0.96346.

10 $$ {w}_{c2}={f}_2\left(w,p\right)=\frac{b_1+{b}_3\cdot w+{b}_5\cdot \ln p+{b}_7\cdot {w}^2+{b}_9\cdot {\ln}^2p+{b}_{11}\cdot w\cdot \ln p}{1+{b}_2\cdot w+{b}_4\cdot \ln p+{b}_6\cdot {x}^2+{b}_8\cdot {\ln}^2p+{b}_{10}\cdot w\cdot \ln p} $$

where *b*_1_ = 1.008, *b*_2_ = 2.618, *b*_3_ = 1.743, *b*_4_ = − 0.808, *b*_5_ = 0.297, *b*_6_ = 2.327, *b*_7_ = 4.605, *b*_8_ = 0.406, *b*_9_ = 0.699, *b*_10_ = − 2.343, *b*_11_ = − 4.984. The correlation coefficient *R*_*2*_ of *f*_*2*_ is 0.95903.

11 $$ {w}_{c3}={f}_3\left(w,p\right)=\frac{c_1+{c}_3\cdot \ln w+{c}_5\cdot p+{c}_7\cdot {\ln}^2w+{c}_9\cdot {p}^2+{c}_{11}\cdot p\cdot \ln w}{1+{c}_2\cdot \ln w+{c}_4\cdot p+{c}_6\cdot {\ln}^2w+{c}_8\cdot {p}^2+{c}_{10}\cdot p\cdot \ln w} $$

where *c*_1_ = 1.279, *c*_2_ = 0.574, *c*_3_ = 0.943, *c*_4_ = − 0.152, *c*_5_ = − 0.291, *c*_6_ = 0.113, *c*_7_ = 0.154, *c*_8_ = 0.009, *c*_9_ = 0.018, *c*_10_ = − 0.062, *c*_11_ = − 0.250. The correlation coefficient *R*_*3*_ of *f*_*3*_ is 0.95244.

**Fig. 1:**
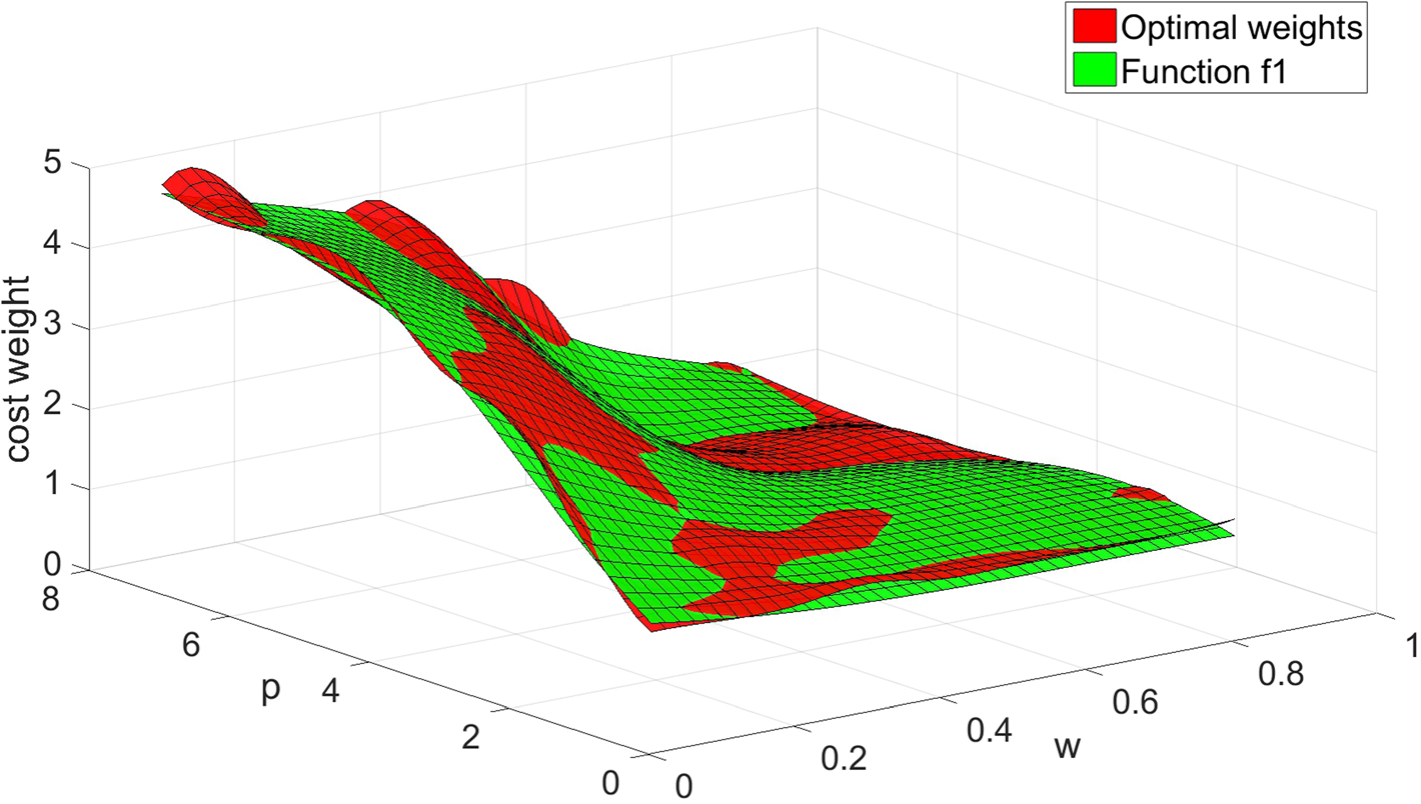
The values of function *w*_*c1*_ compared with the optimal weights

**Fig. 2:**
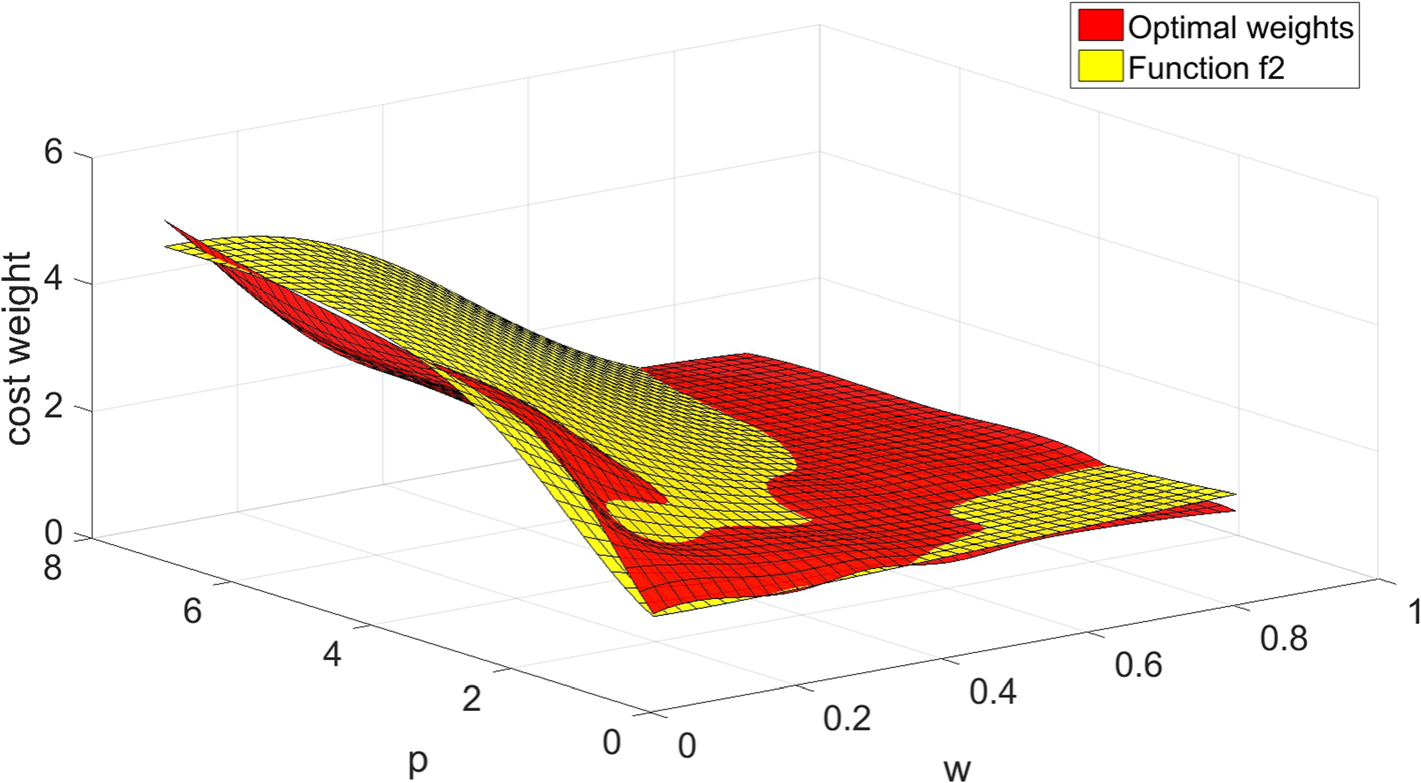
The values of function *w*_*c2*_ compared with the optimal weights

**Fig. 3:**
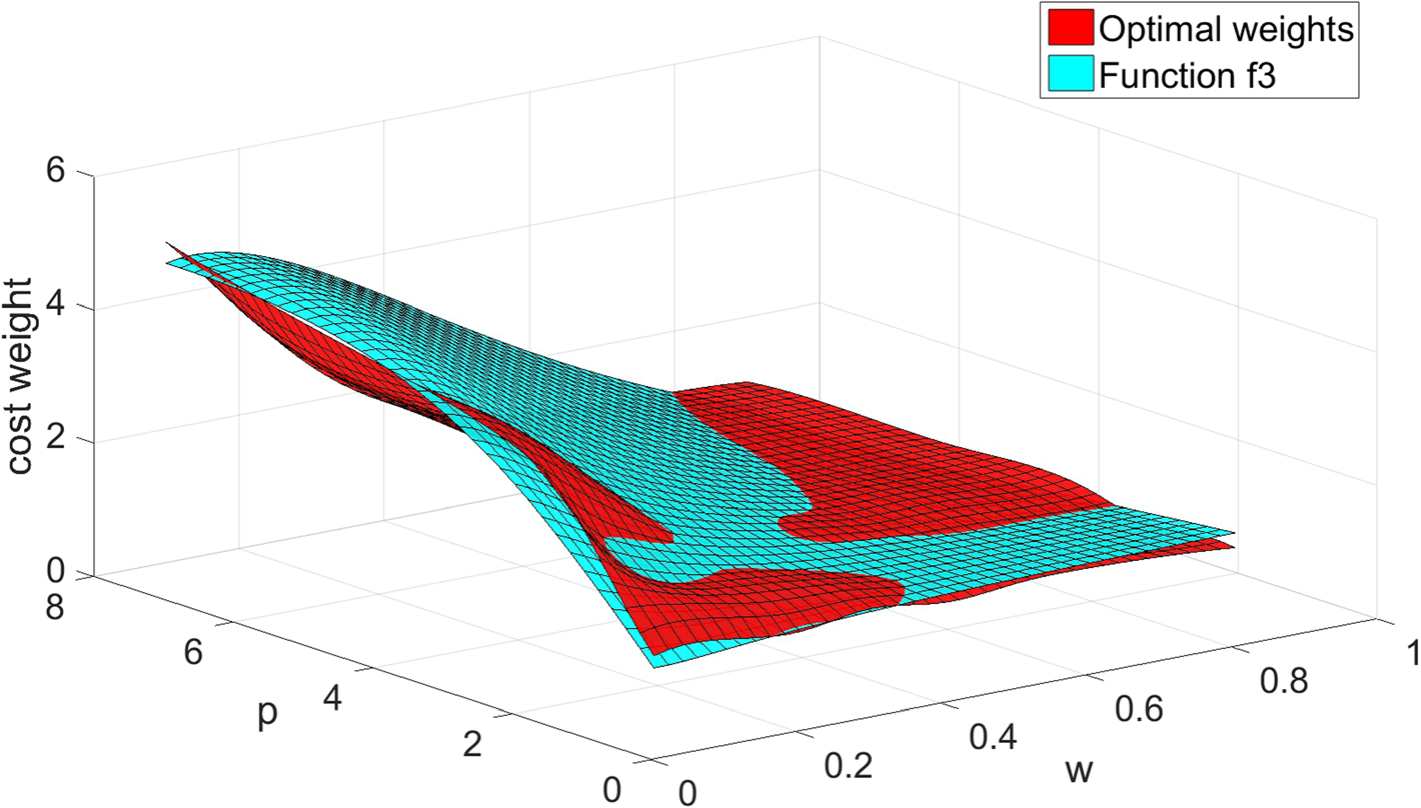
The values of function *w*_*c3*_ compared with the optimal weights

We compare the fitting functions with the optimal weights in Figs. 1, 2 and 3.

Figures 1, 2 and 3 show the comparison results of the three-dimensional interpolation of optimal weights and fitting functions. The red surface represents the optimal weights. The green, yellow, blue planes are fit surfaces of *f*_*1*_, *f*_*2*_ and *f*_*3*_. The correlation coefficient *R* of *f*_*1*_, *f*_*2*_ and *f*_*3*_ identified that the overall fitness of the function *f*_*1*_ is better than other two. The function *f*_*2*_ gradually deviates from optimal weights while we increase the value of *w*, and decrease the value of *p*. The function *f*_*3*_ is slightly coarser than the function *f*_*1*_ in general.

## Discussion

### Comparison with grid searching and function fitting

Using different gene expression datasets, we compared the optimal cost weights obtained from the grid searching strategy and fitted functions *f*_*1*_*, f*_*2*_ and *f*_*3*_. In Table 6, we compared the WCAs with four different datasets, namely, Ovarian, Prostate, Lung1 and Lung2. The majority over minority class proportion of the four datasets are 1.68, 2.5, 5 and 8 respectively. All WCAs are computed using ELM as the base classifier. We also compare the two approaches with ECSELM. The best fit datasets are listed in Table 6.

**Fig. 4:**
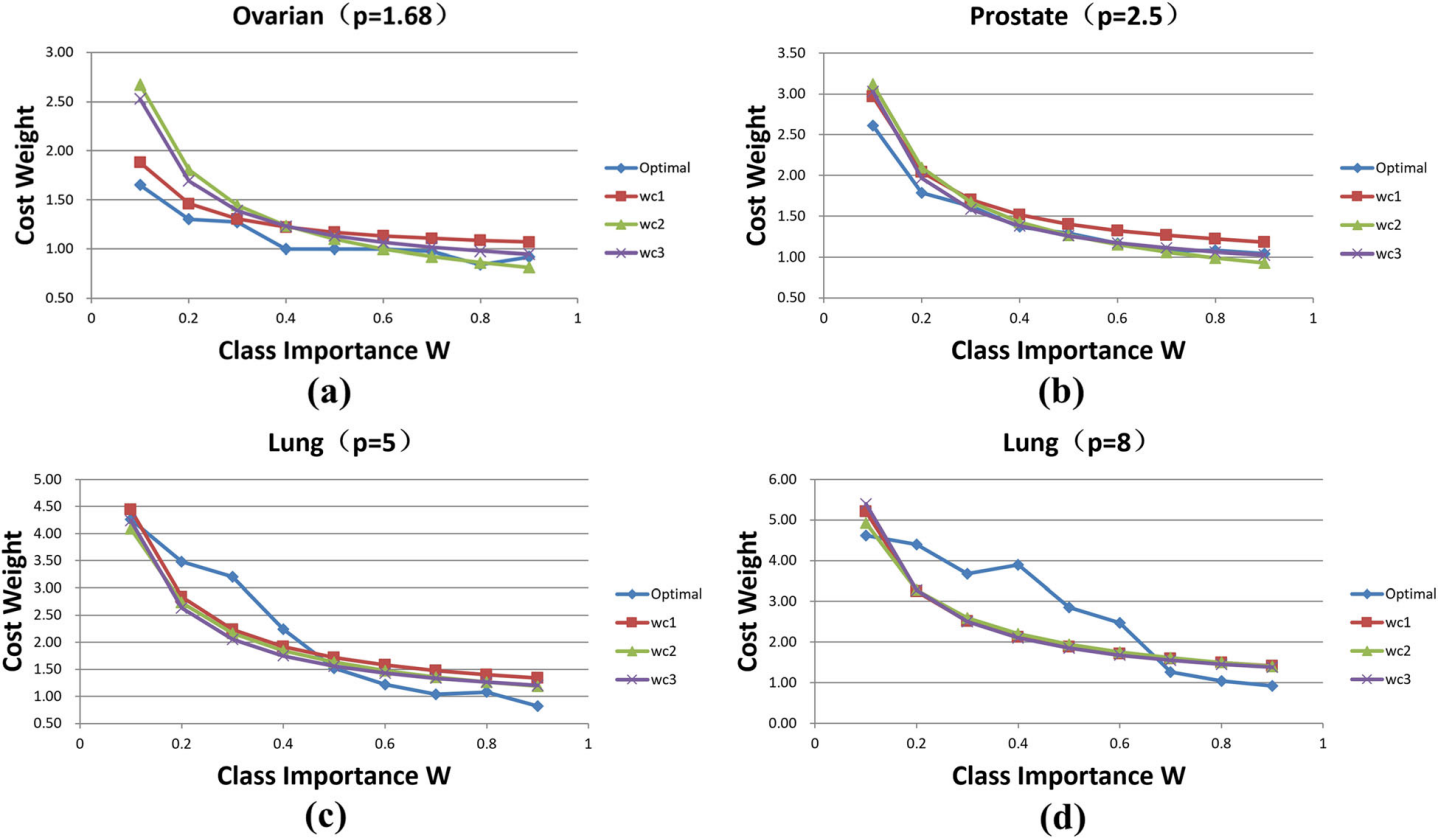
Cost weight comparison using Ovarian, Prostate, Lung1, Lung2 dataset (*p* = 1.68, 2.5, 5, 8)

For each dataset, we plot the weight variance with different values of *w*. For different dataset, the fittest function (choice from *f*_*1*_, *f*_*2*_ and *f*_*3*_) might be different (Fig. 4).

**Fig. 5:**
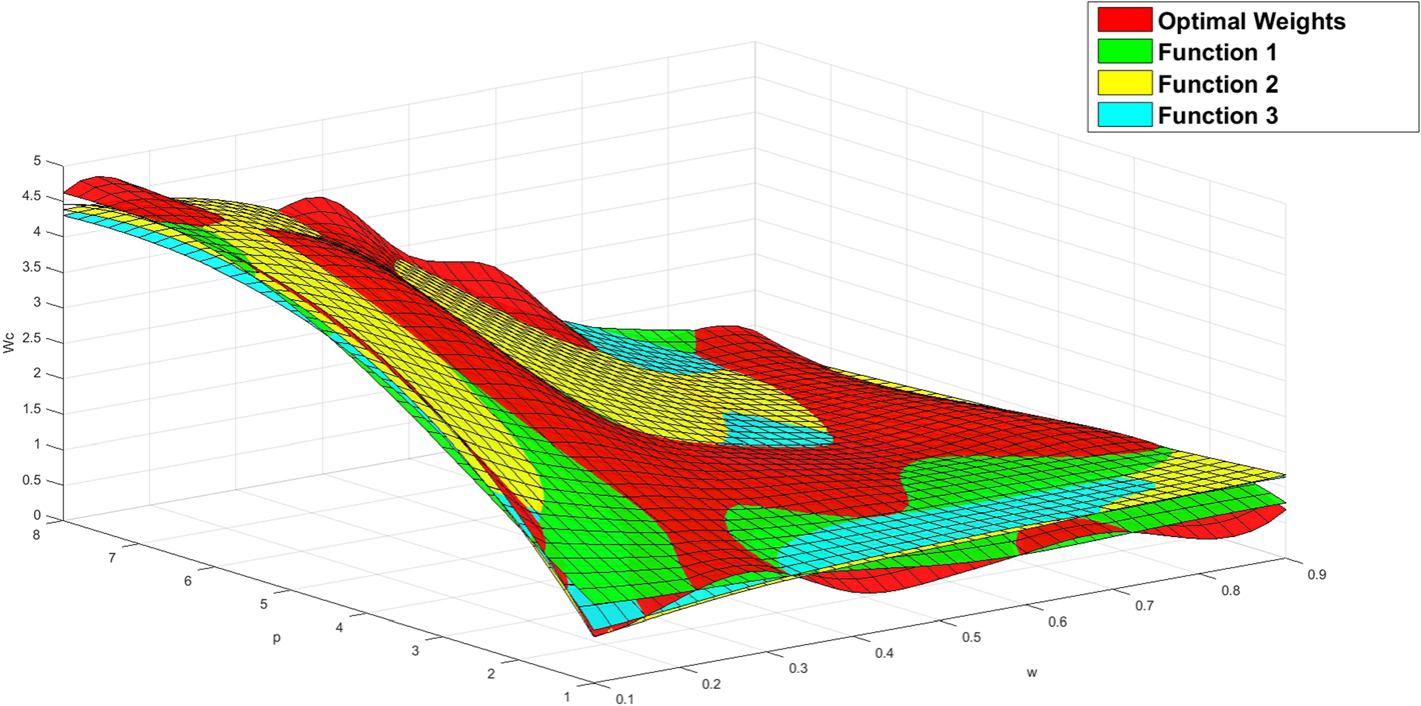
Cost weight comparison in overall

Figure 4 shows that the more unbalanced the dataset is, the higher degree of fitness we can get; and the cost weights obtained from the fitting functions are closer to the optimal weights. In addition, the cost weights from function *f*_*1*_ and *f*_*3*_ are slightly superior to *f*_*2*_. We put all cost weights obtained by different methods in a three-dimensional picture and show the results in Fig. 5.

**Fig. 6:**
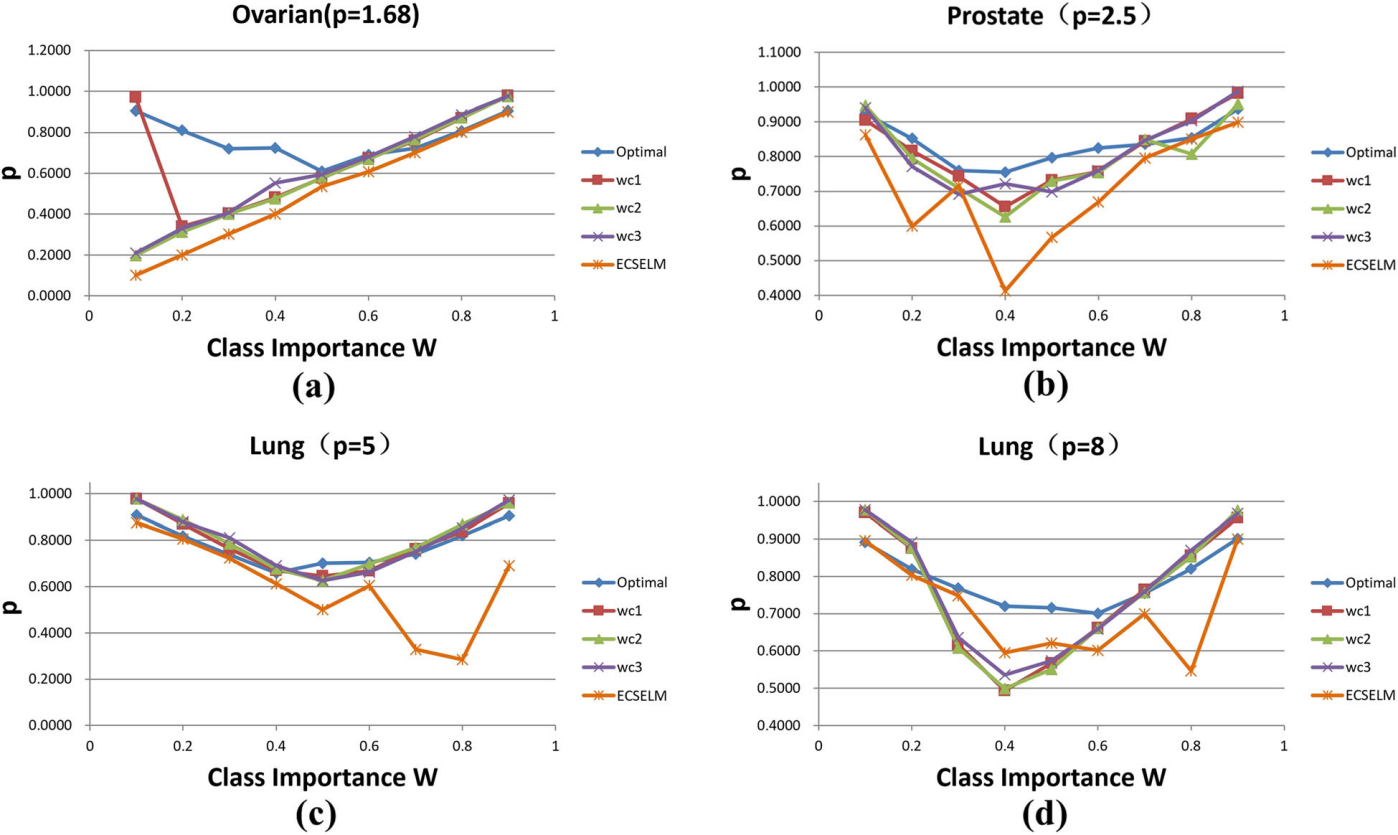
WCA comparison with Ovarian, Prostate, Lung1, Lung2 dataset (*p* = 1.68, 2.5, 5, 8)

For each dataset, we also illustrate the comparison of WCAs against different *w* values (Fig. 6). Besides, we compare WCAs of optimal weights and *f*_*1–3*_ with ECSELM [48].

**Fig. 7:**
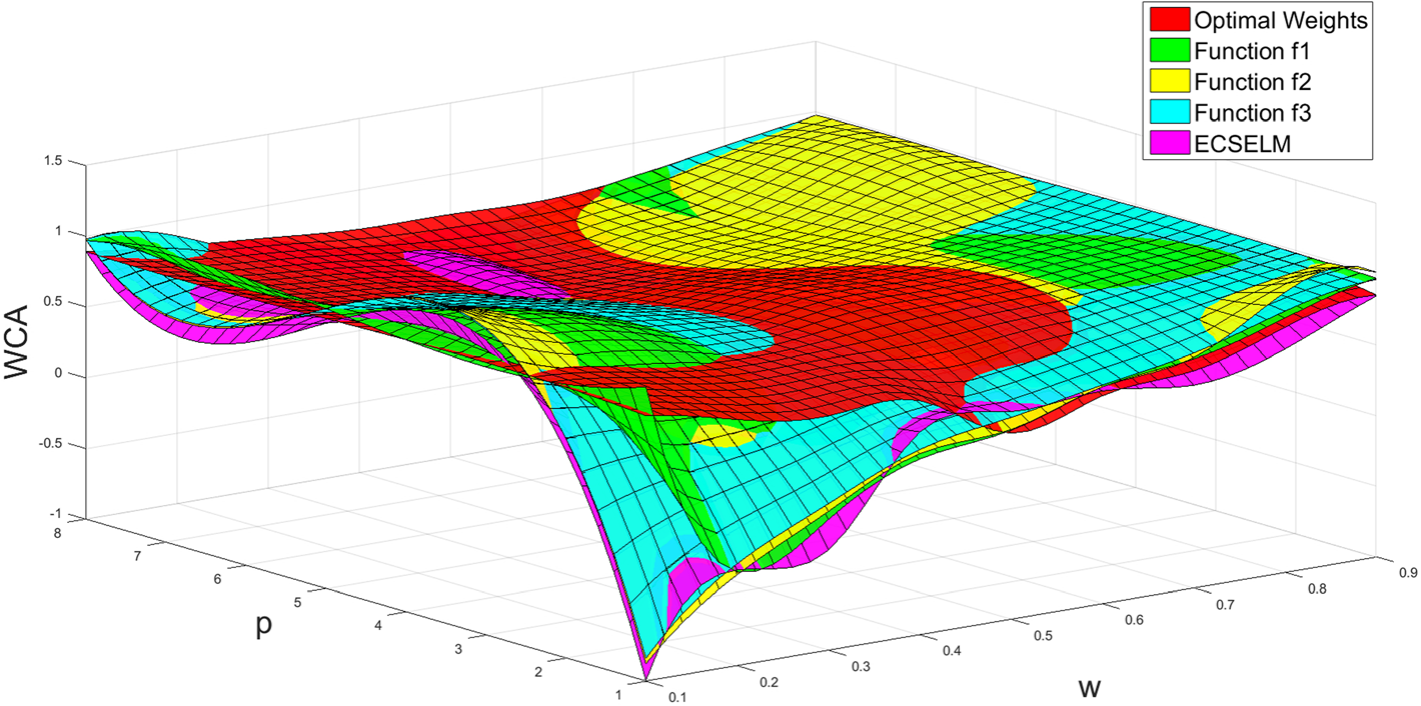
The WCA comparison in 3-dimension

In Fig. 6, we can see that the WCAs of the three fitting functions are lower than the optimal accuracy when *w* is less than 0.5. The reason is that the fitting degree of the cost weights in this range is lower. Moreover, it can be seen from Fig. 6 that the WCAs of the fitting functions approach to the optimal accuracy with the increment of *p*. Furthermore, the WCAs of our approaches is better than ECSELM in most field. Compared with ECSELM, our methods are more stable, and meanwhile can guarantee high WCA. This proves the robustness of our strategy. Similar to the case of cost weights, we ensemble all WCAs obtained by different methods in a three-dimensional picture (Fig. 7). In summary, we find that the function *f*_*1*_ provides better classification performance than the other two functions in general; and the fitting function *f*_*3*_ and *f*_*2*_ have better performance while the valuable *p* is large (when *p* above 5).

## Conclusions

In this paper, we have proposed two approaches to calculate the optimal cost weights for gene expression data. The two approaches include a grid searching strategy and a function fitting method. They enrich the ways of calculating the cost weights for imbalanced data classification problems. In general, the function fitting approach is more efficient than the grid searching strategy. The experimental results also show that the function fitting approach can accurate find the optimal cost weights for imbalanced gene expression datasets.

The limitation of this work is that, although the ELM classifier is tested, the stability of the function fitting method is not proven, especially for other significantly different datasets. The exploration of the proposed algorithm’s stability is left as future work.

## Data Availability

The datasets analyzed in this manuscript are available from http://www.csie.ntu.edu.tw/~cjlin/libsvmtools/datasets/

## Abbreviations

ACA: Adaptive classification accuracy
CCR: Correct classification rates
CSL: Cost sensitive learning
OA: Overall accuracy
WCA: Weighted classification accuracy
